# Effect of Oil Type on Spatial Partition of Resveratrol in the Aqueous Phase, the Protein Interface and the Oil Phase of O/W Emulsions Stabilized by Whey Protein and Caseinate

**DOI:** 10.3390/antiox12030589

**Published:** 2023-02-27

**Authors:** Yang Chen, Hao Cheng, Li Liang

**Affiliations:** 1 State Key Laboratory of Food Science and Technology, Jiangnan University, Wuxi 214122, China; 2 School of Food Science and Technology, Jiangnan University, Wuxi 214122, China; 3 International Joint Laboratory on Food Safety, Jiangnan University, Wuxi 214122, China

**Keywords:** emulsion, spatial partition, whey protein, caseinate, oil type, resveratrol

## Abstract

Oil-in-water emulsions contain the inner oil phase, the protein membrane at the interface and the aqueous phase. In this study, the spatial partition of resveratrol was investigated in sunflower oil, fish oil, medium-chain triglyceride (MCT) and peppermint oil emulsions stabilized by native whey protein isolate (WPI), heat-denatured WPI and sodium caseinate. Resveratrol was added in the aqueous phase of emulsions and its partition was analyzed in term of resveratrol solubility in bulk oil and in the aqueous phase of protein, protein concentration and interfacial protein. The final concentrations of resveratrol in the aqueous phase were basically greater than those in the oil phase of fish oil, sunflower oil and MCT oil emulsions, while the final concentrations of resveratrol in the oil phase were greater than those in the aqueous phase of peppermint oil emulsions. The difference in the interfacial partition of resveratrol and proteins increased as the polyphenol solubility in bulk oil increased. Resveratrol solubility in the oil phase drove its transfer from the aqueous phase into the oil phase in all emulsions, except that the interfacial protein also contributed to the transfer in fish oil emulsions. The oil–water interface provided the microenvironment for the enrichment of resveratrol by proteins.

## 1. Introduction

Oil-in-water (O/W) emulsions contain the oil droplets in the continuous aqueous phase. Proteins emulsions are the constitution of food products such as milk and ice cream and have been used as encapsulate bioactive components for improving their stability, controlled release, absorption and target [1,2,3]. In emulsions, antioxidant activity is affected by the partition of protein and bioactive components [4]. Since oil oxidation mainly occurs at the interface, the interfacial antioxidants are more efficient to improve the chemical stability of emulsions than those in the aqueous phase [5]. Moreover, encapsulation efficiency, digestion stability and bioaccessibility of polyphenols in emulsions were affected by oil type, due to their different distribution into the oil phase [6]. It is thus important to clarify the spatial partition of bioactive components in emulsions for both utilization in foods and functional foods.

Bioactive components can be added in the oil phase or in the aqueous phase of emulsions. When polyoxyethylene-(20)-sorbitanmonooleate (Tween 80) was used as an emulsifier, the partition of antioxidants was related to their hydrophilic–lipophilic balance and aggregation according to the “cut-off” effect [7,8]. However, the prediction of antioxidants’ partition in the interfacial region was unreliable based on their hydrophobicity, since their oil–water partition coefficients might be different from the reported ones in 1-octanol/water mixture [9]. In protein emulsions, the polyphenols at the interface were considered as those in the emulsified oil droplets, without excluding the portion of polyphenols in the oil phase [10,11]. We recently found that resveratrol partition was also dependent on oil type, and the polyphenol could migrate into the oil phase of peppermint oil emulsions [10,12]. It is thus necessary to systematically study the impact of proteins and oils on spatial partition of polyphenols in emulsions.

Resveratrol, a polyphenolic antioxidant, may possibly partition into the oil phase, the interfacial membrane, the protein particles in the aqueous phase and be free in the aqueous phase of O/W emulsions (Figure S1). Tween 20 is a nonionic surfactant, and its ability to competitively replace interfacial β-lactoglobulin and myofibrillar protein has been verified [13,14]. The aim of this study is to analyze the spatial partition of resveratrol in the emulsions made with different oils and proteins. The partition of resveratrol in sunflower oil, fish oil, medium-chain triglyceride (MCT) and peppermint oil emulsions was measured by a combination of centrifugation and interfacial protein replace. The effect of proteins including whey protein isolate (WPI), heat-denatured WPI (hWPI) and sodium caseinate (SC) on the polyphenol partition were also investigated. The polyphenol partition was discussed to find out the empirical formula by analyzing its relationship with the interfacial protein percentage and the polyphenol solubility in the oils and in the aqueous solutions of proteins. The results will be a basis for the potential utilization of protein emulsions.

## 2. Materials and Methods

### 2.1. Materials

Sodium caseinate, polydatin (HPLC grade, >95%) were purchased from Sigma-Aldrich Co. (St. Louis, MO, USA). WPI was purchased from Davisco International, Inc. (Russel, MN, USA). Tween 20 (Biotechnology grade, ≥99%), resveratrol (trans-isomer, ≥98%), fish oil and peppermint oil were respectively obtained from Macklin Co. (Shanghai, China), Sango Biotech Co. (Shanghai, China), Chaopu Co. (Shanghai, China), Ltd. and Zixin Biotechnology Co., Ltd. (Shanghai, China). Sunflower oil (Duoli brand) was purchased from a local market (Wuxi, China). MCT (C8:C10 = 60:40) was purchased from Yong sheng Industry and Trade Co., Ltd. (Guangzhou, China). Other materials of analytical grade were purchased from Sino-Pharm CNCM Ltd. (Shanghai, China).

### 2.2. Emulsion Preparation

SC and WPI at 1–4% (*w*/*w*) were dissolved in Milli-Q ultrapure water and adjusted to pH 7.0 with 0.1 M NaOH or HCl. The solution of hWPI was obtained by heating WPI solution at 90 °C for 35 min. Resveratrol at 50.5 mg/mL was dissolved in 70% (*v*/*v*) ethanol. The ethanol solution of resveratrol was added into protein aqueous solution by stirring for 30 min and diluted with water. The protein–resveratrol aqueous solution was mixed with bulk oil by shearing at 10,000 rpm for 1 min using a high-speed mixer (IKA Ltd., Guangzhou, China) and at 10 °C and 500 bar 3 times using a high pressure homogenizer (ATS Engineering Ltd., Brampton, ON, Canada) [10]. The final emulsions contained 0.50%, 1.0% and 2.0% (*w*/*w*) protein, 10% oil, and 0.13 and 0.26 mg/mL resveratrol.

### 2.3. Particle Size and ζ-Potential

After samples were diluted 200–250 times with pH 7.0 water, size distribution and ζ-potential were determined at 25 °C using NanoBrooker Omni nano particle size analyzer (Brookhaven Instruments Ltd., New York, NY, USA) with a laser diffraction angle of 90°. The NNLS model was used for the analysis of size distribution, while the Smoluchowski model through phase analysis light-scattering (PALS) measurement was used for ζ-potential analysis.

### 2.4. Interfacial Protein Percentage

Emulsions were centrifuged twice at 13,000× *g* for 1 h at 4 °C using a 5804 R centrifuge (Eppendorf Co., Ltd., Hamburg, Germany). The content of protein in the whole emulsion and the supernatant was determined using the Kjeldahl method. Exactly 5 mL of sample was fully digested by mixing with 0.15 g of copper sulfate, 3.0 g of potassium sulfate pentahydrate and 10 mL of concentrated sulphuric acid and heated in graphite digester. The digested mixture was distilled using K9840 automatic Kjeldahl nitrogen determinator (Hanon, Jinan, China) and titrated with 0.05 M HCl. The interfacial protein percentage was calculated using the difference between the protein content in the whole emulsion and that in the aqueous phase divided by the protein content in the whole emulsions [3,10].

### 2.5. Competitive Replacement of Interfacial Protein

After emulsions were centrifuged twice at 13,000× *g* for 1 h at 4 °C to separate the emulsified oil droplets from the continuous phase, the protein at the oil–water interface was competitively replaced by Tween 20 and transferred to the continuous phase [15]. The cream layer was mixed with 3% (*w*/*w*) Tween 20 or water (control) at the corresponding mass of the continuous phase. The mixture was oscillated for 10 s and stirred for 0–2 h. After the mixture was then centrifuged, protein in the mixture and in the supernatant was determined using the Kjeldahl method as mentioned in 2.4. The replacement degree was calculated using the protein in the supernatant divided by the total protein in the mixture.

### 2.6. Partition of Resveratrol in Emulsion

**Figure 1:**
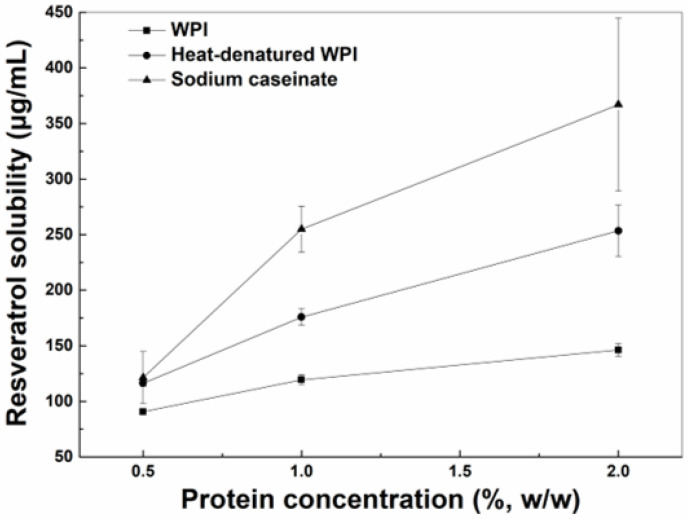
Solubility of resveratrol in the aqueous solution with WPI, heat-denatured WPI and sodium caseinate as a function of protein concentration.

To clarify the spatial partition of resveratrol in Figure 1, the polyphenol in the whole emulsion (*R*_*total*_) and in the supernatant (*R*_*aqueous*_) after double centrifugation at 13,000× *g* for 1 h at 4 °C was measured; the difference between them was recorded as resveratrol in the emulsified oil droplets (*R*_*droplet*_) including the polyphenol in the oil phase (*R*_*oil*_) and at the interface (*R*_*interface*_). The supernatant was adjusted to the isoelectric point of proteins (WPI at pH 5.2, hWPI at pH 5.1 and SC at pH 4.7) and double centrifuged at 13,000× *g*; resveratrol in the serum was recorded as free resveratrol (*R*_*free*_) in the continuous phase. *R*_*aqueous*_ is the sum of *R*_*free*_ and the polyphenol encapsulated in protein particles of the aqueous phase. Resveratrol at the interface (*R*_*interface*_) of the emulsified oil droplets was separated by the replacement of Tween 20. The partition of resveratrol in emulsion was calculated as follows

(1) $$\mathrm{Percentage}\;\mathrm{of}\;\mathrm{resveratrol}\;\mathrm{in}\;\mathrm{the}\;\mathrm{aqueous}\;\mathrm{phase}\;(\%)=\frac{R_{aqueous}}{R_{total}}\times 100$$

(2) $$\mathrm{Percentage}\;\mathrm{of}\;\mathrm{resveratrol}\;\mathrm{free}\;\mathrm{in}\;\mathrm{the}\;\mathrm{aqueous}\;\mathrm{phase}\;(\%)=\frac{R_{free}}{R_{total}}\times 100$$

(3) $$\mathrm{Percentage}\;\mathrm{of}\;\mathrm{resveratrol}\;\mathrm{at}\;\mathrm{the}\;\mathrm{interface}\;(\%)=\frac{R_{interface}}{R_{total}}\times 100$$

(4) $$\mathrm{Percentage}\;\mathrm{of}\;\mathrm{resveratrol}\;\mathrm{in}\;\mathrm{the}\;\mathrm{oil}\;\mathrm{phase}\;(\%)=\frac{R_{droplet}-R_{interface}}{R_{total}}\times 100$$

### 2.7. Quantitation of Resveratrol Using HPLC and UV Method

Resveratrol was extracted and analyzed according to our previous report [16,17]. Samples were vortex-oscillated with methanol at a volume ratio of 1:10 for 1 min and centrifuged at 10,000× *g* at 4 °C. For UV method, resveratrol in the supernatants was detected at 306 nm using a Synergy H1 microplate reader (Agilent Co., Ltd., New York, NY, USA). The sample without resveratrol was used as a blank. For HPLC methods, 0.2 mM polydatin (internal standard) in methanol was added in samples. After centrifugation, the supernatants were injected into the HPLC system with a 2998 PDA detector and a C18 column (5 μm, 4.6 mm × 250 mm, Waters, Milford, MA, USA) at the column temperature of 35 °C. The mobile phase was an isocratic mixture of methanol and Milli-Q water (50:50, *v*/*v*) at a flow rate of 1 mL·min^−1^.

### 2.8. Solubility of Resveratrol in Protein Solution and Bulk Oil

Resveratrol at 50.5 mg/mL in 70% ethanol was added into the aqueous solution of proteins in a volume ratio of 1:100 and shaken at 25 °C for 2 h. After the mixture was centrifuged at 4000× *g* for 15 min, the supernatant was diluted with Milli-Q water for absorbance measurement, and the protein blank solutions without resveratrol were used as control. The solubility of resveratrol was determined using an external standard method.

The solubility of resveratrol in bulk oil was determined according to the method of [18]. The excess resveratrol powder was dispersed in bulk oils by shaking at 37 °C for 24 h and centrifuged at 10,000× *g* and 4 °C for 30 min. Methanol was then added to the upper phase by vortexing for 1 min and re-centrifuged for 10 min. The supernatant was collected to quantify resveratrol using HPLC. Bulk oils without resveratrol were used as a control.

### 2.9. Statistical Analysis

All the results were repeated at least three times and presented as mean values ± standard deviations. Statistical analysis was performed using SPSS (IBM Co., Ltd., New York, NY, USA).

## 3. Results and Discussion

### 3.1. Solubility of Resveratrol in Bulk Oils and Protein Aqueous Solutions

The solubility of resveratrol was ranked in the order of peppermint oil > MCT > fish oil > sunflower oil (Table 1). Fish oil is rich in long-chain unsaturated fatty acids. The content of unsaturated fatty acids is 85–91% in sunflower oil [19]. MCT is a saturated fatty acid with a carbon chain length of 8–12. Peppermint oil is a mixture of alcohols, ketones, esters and terpenes, with menthone and menthol accounting for more than 60% of the total [20]. The solubility of resveratrol in the oils is consistent with the oil dielectric constants (Table 1). It is easier for the more polar groups in the oil phase to induce dipole–dipole interactions between hydroxyl groups on resveratrol and fatty acid polar groups [21].

The solubility of resveratrol in the aqueous solutions increased as the concentrations of WPI, hWPI or SC increased from 0.5% to 2% (Figure 1). The solubility of resveratrol in the presence of SC or hWPI was greater than that in the presence of WPI at 0.5%, while the polyphenol solubility in protein solutions ranked in the order of SC > hWPI > WPI at higher concentrations. The increase in the solubilization of resveratrol may be due to the polyphenol binding to proteins. The interaction between proteins and resveratrol was mainly driven by hydrogen bond and hydrophobic interactions, and the polyphenol binding constants with WPI and SC were, respectively, 1.2 × 10^5^ M^−1^ and (3.7–5.1) × 10^5^ M^−1^ [22,23]. The loading efficiencies of resveratrol in SC particles were reportedly greater than those in WPI particles when the protein concentration was 1% [24]. Thermal denaturation caused exposure of more hydrophobic residues, improving the affinity of resveratrol to β-lactoglobulin, a major whey protein [25].

### 3.2. Interfacial Protein in Emulsions

**Figure 2:**
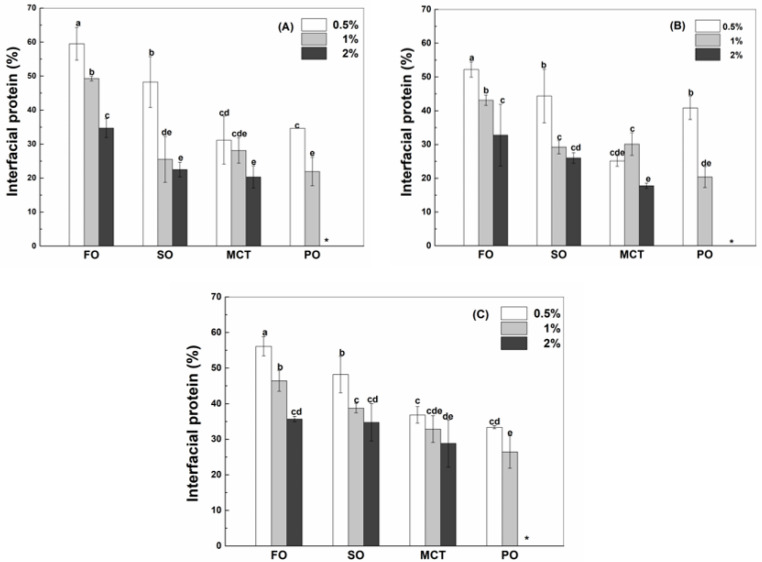
Interfacial percentage of native WPI (**A**), heat-denatured WPI (**B**) and sodium caseinate (**C**) in sunflower oil, fish oil, MCT and peppermint oil emulsions with 130 μg/mL resveratrol. Protein concentrations are 0.5%, 1% and 2%. Different letters mean significant differences at *p* < 0.05. * Peppermint oil emulsions were unstable at 2% proteins.

The interfacial protein percentage decreased as the concentration of WPI (Figure 2A), hWPI (Figure 2B) or SC (Figure 2C) increased from 0.5% to 2% in emulsion, which was not significant for sunflower oil emulsions with 1% and 2% proteins and MCT emulsions with 0.5% and 1% proteins. The interfacial percentages of WPI, hWPI and SC at the same concentration were similar in fish oil and peppermint oil emulsions (Figure 2). In sunflower oil emulsions, the interfacial percentages of WPI, hWPI and SC were similar at 0.5%, while the interfacial percentages of SC (Figure 2C) were greater than those of WPI (Figure 2A) and hWPI (Figure 2B) at higher protein concentrations. These results are consistent with 20% soya oil emulsions stabilized by WPI and caseinate at pH 7, where WPI and caseinate adsorbed to the oil–water interface at the same extent at low concentrations, but caseinate adsorbed in preference to WPI with an excess of proteins [26]. In MCT emulsions, the interfacial percentages of SC (Figure 2C) were greater than those of WPI (Figure 2A) and hWPI (Figure 2B) at 0.5–2%. The protein layer at the oil–water interface is in a dynamic equilibrium which could be affected by the structure and intermolecular interaction of proteins [27]. The protein layer will undergo reversible collapse when the amount of protein exceeds the maximum molecule density, and the reform of the interfacial membrane was driven by the attraction force of protein at the oil–water interface [28]. The peppermint oil emulsions with 2% proteins were not analyzed, since they separated into the creaming layer and the aqueous phase upon preparation with SC and after 2 days with WPI and hWPI.

Oil type affects the adsorption of proteins at the oil–water interface (Figure 2). When the protein content was 0.5%, the interfacial percentages of WPI or SC in fish oil and sunflower oil emulsions were greater than those in MCT and peppermint oil emulsions (Figure 2A,C), while the percentage of hWPI decreased in the sequence of fish oil, sunflower oil, peppermint oil and MCT emulsions (Figure 2B). At 1% proteins, the interfacial percentages of WPI, hWPI or SC ranked in the order of fish oil > sunflower oil ~ MCT > Peppermint oil (Figure 2). In the case of 2% proteins, the percentage of WPI in fish oil emulsions was greater than those in sunflower oil and MCT emulsions (Figure 2A); the percentage of hWPI ranked in the order of fish oil > sunflower oil > MCT (Figure 2B), and the percentages of SC in fish oil and sunflower oil emulsions were greater than that in MCT emulsions (Figure 2C). The polarity of four oils was ranked in the order of peppermint oil > MCT > fish oil ~ sunflower oil (Table 1). It has been reported that protein adsorption was slower on the surface of more polar oils. The expansion and adsorption of proteins were higher on the surface of hydrophobic oils, while proteins were adsorbed in a random orientation at a polar oil–water interface with lower interfacial tensions [29].

In 50% walnut oil emulsions stabilized by 4% SC, the loading content of SC at the surface of oil droplets was not affected by 2 mM resveratrol but improved by 4 and 6 mM resveratrol [11]. In this study, the concentration of resveratrol is 130 μg/mL (~0.6 mM). The interfacial percentages of WPI, hWPI and SC were not basically affected by addition of resveratrol at the protein concentration of 1% (Figure S2).

### 3.3. Interfacial Protein Replacement

When fish oil emulsions with 0.5% proteins were centrifuged and their cream layer was mixed with ultra-pure water under vortexing for 30 s, without and with supplemental stirring for 2 h, the interfacial protein replacement was 16–43% (data not shown) in the initial experiments. When walnut oil emulsions were co-stabilized by 0.5% (*w*/*v*) WPI and 0.4% Tween 20, WPI was not detected at the interface, indicating that Tween 20 was more surface-active than WPI [15]. For the effect of Tween 20 on curcumin’s partition in protein particles, it was found that curcumin was encapsulated into the micelle of Tween 20 at 0.2 g/L, suggesting the ability of Tween 20 to capture polyphenols [30]. WPI, hWPI and SC at the interface of fish oil emulsions were completely replaced by 3% Tween 20 under vortexing for 30 s, without and with supplemental stirring for 2 h (Figure S3A). In the case of fish oil, sunflower oil, MCT and peppermint oil emulsions stabilized by 2% proteins, WPI, hWPI and SC were also basically replaced by Tween 20 under vortexing for 30 s (Figure S3B). Therefore, the protein replacement by Tween 20 under vortexing for 30 s was used to separate resveratrol at the oil–water interface from that in the inner oil phase of protein-emulsified oil droplets.

### 3.4. Spatial Partition of Resveratrol in Emulsions

**Figure 3:**
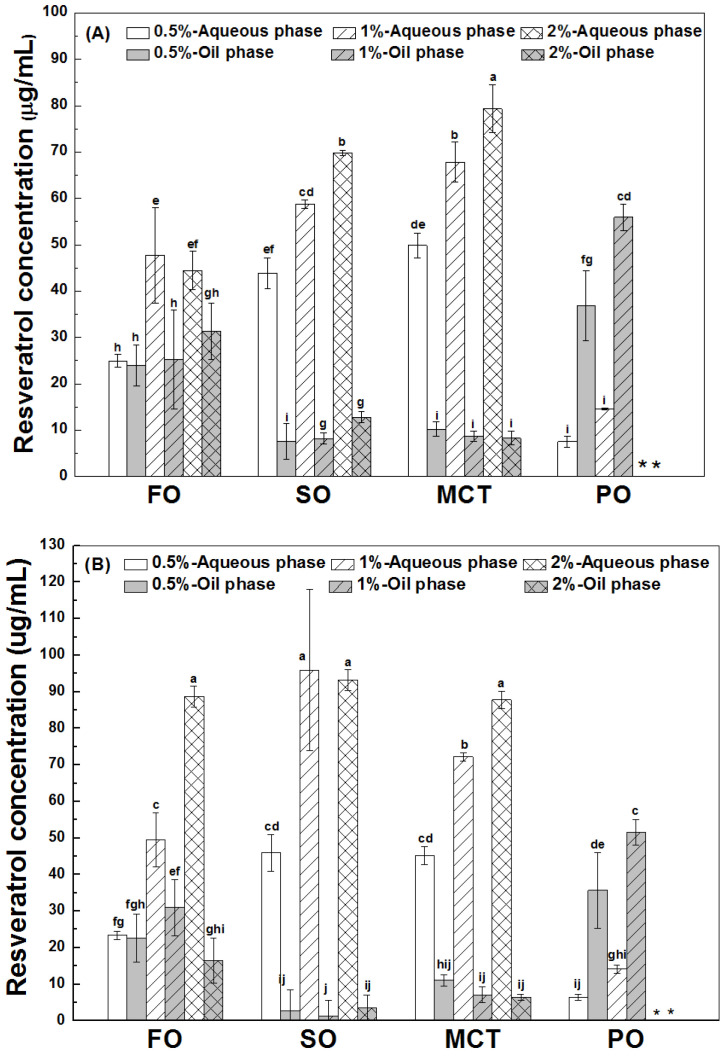

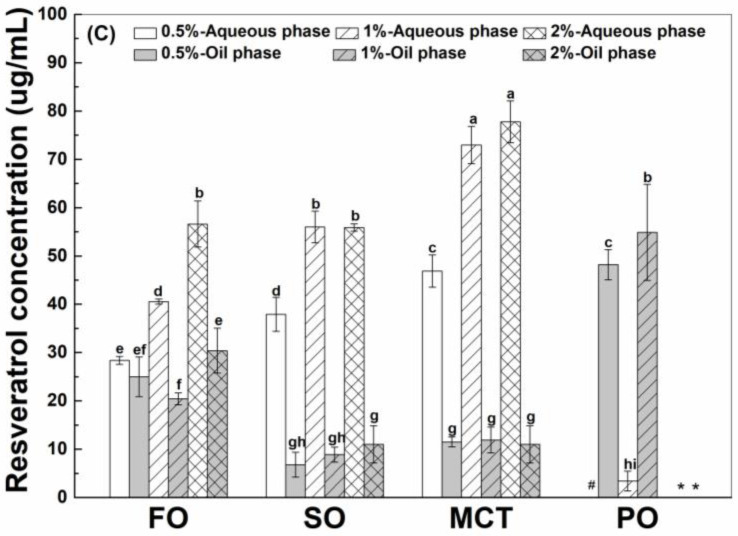
Final concentration of resveratrol in the aqueous and oil phase of emulsions stabilized by WPI (**A**), heat-denatured WPI (**B**) and sodium caseinate (**C**) at 0.5%, 1% and 2%. Different letters mean significant differences at *p* < 0.05. * Peppermint oil emulsions were unstable at 2% proteins. # Resveratrol was not detected.

As shown in Figure 3, the final concentrations of resveratrol in the aqueous phase were greater than those in the oil phase of fish oil, sunflower oil and MCT oil emulsions, except for similar concentrations of resveratrol in the aqueous and oil phase of fish oil emulsions stabilized by 0.5% proteins. However, the concentrations of resveratrol in the oil phase were greater than those in the aqueous phase of all peppermint oil emulsions. These results suggest the transfer of resveratrol from the aqueous phase into the inner oil phase of emulsions. The difference between resveratrol concentrations in the aqueous and oil phase suggests that the protein interface plays an important role for the polyphenol partition in emulsions.

#### 3.4.1. Resveratrol in the Aqueous Phase

**Figure 4:**
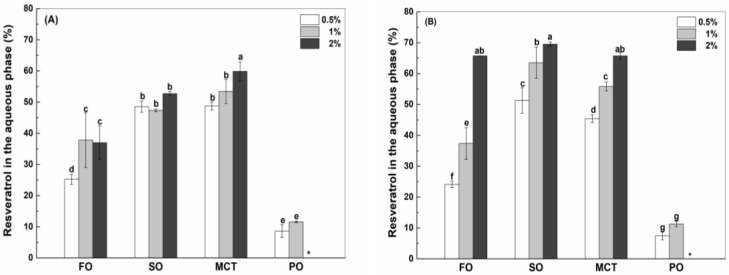

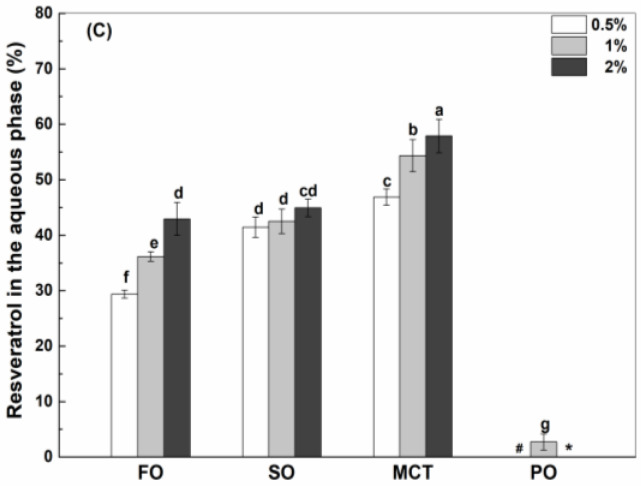
Percentage of resveratrol in the aqueous phase of fish oil (FO), sunflower oil (SO), MCT and peppermint oil (PO) emulsions stabilized by native WPI (**A**), heat-denatured WPI (**B**) and sodium caseinate (**C**) at 0.5%, 1% and 2%. Different letters mean significant differences at *p* < 0.05. * Peppermint oil emulsions were unstable at 2% proteins. # Resveratrol was not detected.

In the aqueous phase of emulsions, resveratrol might be in the free state and encapsulated in the protein particles (Figure S1). Due to the low solubility of resveratrol in water (~30 μg/mL, [31]), the percentages of free resveratrol in the aqueous phase were only around 5% in sunflower oil, fish oil and MCT emulsions stabilized by 2% protein, which were independent of protein type (Figure S4). Free resveratrol was not detected in the aqueous phase of peppermint oil emulsions stabilized by proteins. In peppermint oil emulsions, resveratrol was negligible in the aqueous phase of SC-stabilized emulsions (Figure 4C), while the percentages of resveratrol were around 10% in the aqueous phase of WPI and hWPI emulsions, which was independent of the protein concentrations (Figure 4A,B).

For the use of WPI as an emulsifier, the percentage of resveratrol in the aqueous phase of fish oil emulsions increased from 25% at the protein concentration of 0.5% to 37% at the protein concentrations of 1% and 2% (Figure 4A). The aqueous percentages of resveratrol in sunflower oil emulsions were about 49%, which is independent on WPI concentration. The aqueous percentage of resveratrol in MCT emulsions increased from 49% to 60% as WPI concentration increased from 0.5% to 2%. For comparison, the aqueous percentages of resveratrol in fish oil emulsions were less than those in sunflower oil and MCT emulsions. In the case of hWPI, the aqueous percentage of resveratrol increased from 24% to 66% in fish oil emulsions, from 51% to 69% in sunflower oil emulsions and from 45% to 65% in MCT emulsions, as the protein concentration increased from 0.5% to 2% (Figure 4B). For comparison, the aqueous percentages of resveratrol in fish oil emulsions were less than those in sunflower oil and MCT emulsions at the concentration of hWPI being 0.5% but were similar at 2% hWPI. In the case of SC, the aqueous percentage of resveratrol in fish oil and MCT emulsions increased from 29% to 43% and from 47% to 58%, respectively, as the protein concentration increased from 0.5% to 2% (Figure 4C). The percentage of resveratrol in sunflower oil emulsions were 43%, independent of the concentration of SC. For comparison, the aqueous percentages of resveratrol were ranked in the order of fish oil < sunflower oil < MCT.

When the concentration of proteins was 0.5%, the percentage of resveratrol in the aqueous phase of SC-stabilized fish oil emulsions (Figure 4C) was greater than those in WPI and hWPI-stabilized ones (Figure 4A,B), while the reverse was observed in sunflower oil emulsions. The aqueous percentages of resveratrol were independent of protein type in MCT emulsions (Figure 4A–C). When the concentration of proteins was 2%, the percentage of resveratrol in the aqueous phase of hWPI (Figure 4B) was greater than the percentages in WPI and SC (Figure 4A,C) for fish oil, sunflower oil and MCT emulsions.

#### 3.4.2. Interfacial Resveratrol

**Figure 5:**
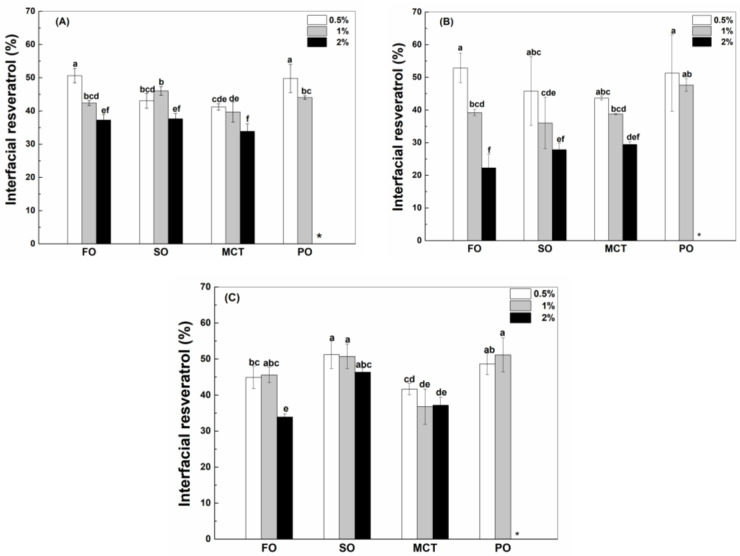
Interfacial percentage of resveratrol in fish oil (FO), sunflower oil (SO), MCT and peppermint oil (PO) emulsions stabilized by native WPI (**A**), heat-denatured WPI (**B**) and sodium caseinate (**C**) at 0.5%, 1% and 2%. Different letters mean significant differences at *p* < 0.05. * Peppermint oil emulsions were unstable at 2% proteins.

Oil type affects the interfacial percentage of resveratrol in emulsions (Figure 5). When the concentration of WPI and hWPI was 0.5%, the interfacial percentages of resveratrol in fish oil and peppermint oil emulsions were greater than those in sunflower oil and MCT emulsions (Figure 5A,B). The interfacial percentages of resveratrol were between 39% and 46% in the emulsions with 1% WPI (Figure 5A), while the interfacial percentages in peppermint oil emulsions were greater than those in fish oil, sunflower oil and MCT emulsions with 1% hWPI (Figure 5B). The interfacial polyphenol percentages were similar in all the emulsions stabilized by 2% WPI and hWPI (Figure 5A,B). In the case of 0.5–2% SC, the interfacial percentages of resveratrol in sunflower oil and peppermint oil emulsions were basically greater than those in fish oil emulsions and MCT emulsions (Figure 5C).

Protein type affects the interfacial percentage of resveratrol (Figure 5). In fish oil emulsions, the interfacial percentage was greater in the presence of WPI (Figure 5A) and hWPI (Figure 5B) than SC (Figure 5C) at 0.5% and in the presence of WPI and SC than hWPI at 1% and 2%. In sunflower oil emulsions, the interfacial percentage was ranked in the order of SC > WPI > hWPI at 0.5–2%. In MCT emulsions, the interfacial percentages of resveratrol were similar in the presence of WPI, hWPI and SC at 0.5% and 1% but greater in the presence of SC and WPI than hWPI at 2%. In peppermint oil emulsions, the interfacial percentages were similar in the presence of WPI, hWPI and SC at 0.5% but ranked in the order of SC > hWPI > WPI at 1%.

It was previously considered that resveratrol was complexed or encapsulated by proteins and then adsorbed together with proteins to the oil–water interface, due to the low solubility of resveratrol in the oils rich in long-chain triglycerides [32,33]. By comparative analysis, the interfacial percentages of resveratrol (Figure 5B) were similar to that of hWPI (Figure 2B) at 0.5–2% in sunflower oil emulsions. In fish oil emulsions, the interfacial percentages of resveratrol (Figure 5A,C) were greater than those of WPI and SC at 0.5% but became similar to those of WPI and SC at 2% (Figure 2A,C), while the interfacial percentages of resveratrol (Figure 5B) were similar to those of hWPI at 0.5% but became less than those of hWPI at 2% (Figure 2B). The interfacial percentages of resveratrol (Figure 5) were greater than the interfacial percentages of proteins (Figure 2) in MCT and peppermint oil emulsions. Therefore, the difference in the partition of resveratrol and proteins at the oil–water interface increased as the polyphenol solubility in bulk oils increased (Table 1).

#### 3.4.3. Resveratrol in the Oil Phase

**Figure 6:**
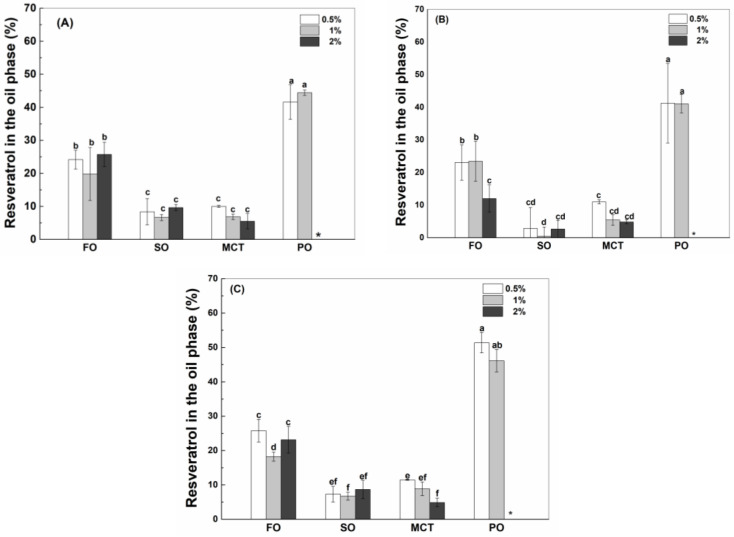
Percentage of resveratrol in the oil phase of fish oil (FO), sunflower oil (SO), MCT and peppermint oil (PO) emulsions stabilized by native WPI (**A**), heat-denatured WPI (**B**) and sodium caseinate (**C**) at 0.5%, 1% and 2%. Different letters mean significant differences at *p* < 0.05. * Peppermint oil emulsions were unstable at 2% proteins.

As shown in Figure 6, the percentages of resveratrol in the oil phase of peppermint oil emulsions were the greatest, which is consistent with the highest polyphenol solubility in peppermint oil (Table 1). The solubility of resveratrol in peppermint oil (Table 1) is much greater than the solubility in the aqueous solution of proteins (Figure 1). When resveratrol in the whole emulsions is hypothetically added to the inner oil phase, its concentration is only 8% of its solubility in peppermint oil. Therefore, the high solubility of resveratrol drives its transfer from the aqueous phase to the inner peppermint oil (Figure 6). It has been reported that the transfer rate of aroma compounds through the interface depends on their affinity toward the liquid phases of a static model system [34].

The percentages of resveratrol in the oil phase of fish oil emulsions were greater than those in the oil phase of sunflower oil and MCT emulsions (Figure 6), which is not consistent with the polyphenol solubility in bulk oils (Table 1). Considering the greater interfacial percentage of proteins in fish oil emulsions than MCT emulsions (Figure 2), it is speculated that the transfer of resveratrol from the aqueous phase into the oil phase also depends on the interfacial partition of protein. The percentages of resveratrol in the phase of fish oil, sunflower oil and MCT were independent of the protein concentration (Figure 6), except for the decrease at a higher protein concentration in hWPI-stabilized fish oil and MCT emulsions (Figure 6B) and SC-stabilized MCT emulsions (Figure 6C). At the same protein concentration, the percentages of resveratrol in the oil phase were independent of protein type (Figure 6), except for the lower percentage in hWPI-stabilized sunflower oil emulsions at 0.5–2% and hWPI-stabilized fish oil emulsions at 2% (Figure 6B). These results suggest that the transfer of resveratrol from the aqueous phase into the oil phase needs to be systematically analyzed in order to make a good prediction of the polyphenol partition in emulsions.

### 3.5. Mechanism of Resveratrol Partition in Emulsions

In the emulsions stabilized by low-molecular-weight surfactants, there was a basic assumption that antioxidants distribute among the oil phase, the aqueous phase and the interfacial region according to their solubilities in each region [35]. The transfer of hydrophilic caffeic acid or catechin from the aqueous phase to the interfacial region was reportedly spontaneous in corn oil emulsions stabilized by Tween 20, when 4-hexadecylbenzenediazonium was used as a chemical probe in the interfacial region [36,37]. It was found that more than 85% of resveratrol located in the interface, and a small fraction in the oil and aqueous regions of corn oil emulsions were stabilized by Tween 20 [38]. In comparison, the interfacial percentages of resveratrol were less in protein-stabilized emulsions (Figure 5).

Multiple linear regression analysis was thus performed to clarify the combined effect of resveratrol solubility in bulk oil (Table 1) and in the aqueous solution of proteins (Figure 1), and protein partition (Figure 2) on resveratrol partition (Figure 4, Figure 5 and Figure 6) in emulsions. The greater the absolute value of the standardized regression coefficient (β), the stronger the dependence on the variables [39]. In fish oil, sunflower oil, MCT and peppermint oil emulsions, there is a negative correlation (*p* < 0.01) between the aqueous percentage of resveratrol with the percentage of interfacial protein (*P*_i_) and the solubility of resveratrol in bulk oils (*R*_o_, Table 2). The β value of *R*_o_ is greater than that of *P*_i_, suggesting that the polyphenol solubility in the oil phase is more important for removing resveratrol in the aqueous phase. The aqueous percentages of resveratrol can be calculated using the optimized Equation (5), where 80.3% of the variability could be accounted for by *R*_o_ and *P*_i_. There was a good correlation between the predicted value by Equation (5) and the experienced value (Figure S5A).

The aqueous percentage = 88.295 − 0.003 × *R*_o_ − 1.018 × *P*_i_ (5)

In fish oil, sunflower oil, MCT and peppermint oil emulsions, there is a positive correlation (*p* < 0.01) between the oily percentage of resveratrol with *P*_i_, *R*_o_ and protein concentration in emulsions (*P*_t_) in Table 3. According to the β values, the importance of the variables was ranked in the order of *R*_o_ > *P*_i_ > *P*_t_. The oily percentages of resveratrol can be calculated using the optimized Equation (6), where 77.9% of the variability could be accounted for by *R*_o_, *P*_i_ and *P*_t_. The content of proteins at the oil–water interface can be calculated by multiplying *P*_i_ and *P*_t_. It is thus suggested that the accessibility of protein to the oil–water interface contributes to the transfer of resveratrol from the aqueous phase into the inner oil phase. When fish oil emulsions were excluded, the correlation between the predicted and experienced values (Figure S5B,D) was improved. It can be seen that the oily percentage of resveratrol is only correlative to Ro (Table 4). The oily percentages of resveratrol in sunflower oil, MCT and peppermint oil emulsions can be calculated using the optimized Equation (7), where 91.2% of the variability could be accounted for by *R*_o_. These results suggest that resveratrol solubility in the oil phase drives its transfer from the aqueous phase into the phase of sunflower oil, MCT and peppermint oil in emulsions, while the combination of resveratrol solubility in the oil phase with interfacial protein contributes to the polyphenol transfer from the aqueous phase into the phase of fish oil in emulsions.

The oily percentage = −30.637 + 0.003 × *R*_o_ + 0.808 × *P*_i_ + 7.994 × *P*_t_ (6)

The oily percentage = 2.114 + 0.002 × *R*_o_ (7)

The oil–water interface has higher interfacial stress in apolar than polar oils, provoking stronger hydrophobic interactions between the oil components and hydrophobic residues of proteins [40]. The polarity of fish oil is the lowest of all oils (Table 1), resulting in a stronger hydrophobic interface and greater adsorption of proteins (Figure 2). The adsorbed protein at the oil–water interface improves the accessibility of protein-loaded resveratrol to the oil phase, contributing to the polyphenol transfer into the inner oil phase. Therefore, the percentages of resveratrol in the fish oil phase were greater than those in the MCT phase of emulsions (Figure 6), although the solubility of resveratrol in MCT was greater than that in fish oil (Table 1). Moreover, the greater loading of resveratrol by SC than by WPI and hWPI (Figure 1) corresponded to greater transfer of resveratrol from the aqueous phase into the oil phase of peppermint oil emulsions (Figure 4 and Figure 6).

In fish oil, sunflower oil, MCT and peppermint oil emulsions, there is a positive correlation regarding the interfacial percentage of resveratrol with *P*_i_ and *R*_o_, but a negative correlation between the interfacial percentage of resveratrol with protein concentration in emulsions (Table 5). Protein concentration in emulsions and *P*_i_ could be considered as one variable since they had a significant negative correlation (Table S1). The interfacial percentage of resveratrol can be calculated using the optimized Equation (8), where R^2^ indicates that 52.1% of the variabilities could be accounted for by *P*_i_ and *R*_o_ (Table 5). The correlation between the predicted and experienced values was improved when sunflower oil emulsions were excluded (Figure S5C,E). The interfacial percentages of resveratrol can be calculated using the optimized Equation (9), where 71.5% of the variability could be accounted for by *R*_o_ and *P*_i_ (Table 6). *R*_o_ and *P*_i_ have close β value values, suggesting both factors are important for the interfacial partition of resveratrol in emulsions.

The interfacial percentage = 21.351 + 0.001 × *R*_o_ + 0.533 × *P*_i_ (8)

The interfacial percentage = 17.146 + 0.001 × *R*_o_ + 0.566 × *P*_i_ (9)

The complexation with resveratrol had no impact on the adsorption of proteins at the interface (Figure S2). Additionally, the interfacial resveratrol (Figure 5) is greater than the interfacial proteins (Figure 3), of which the difference increased as the polyphenol solubility in bulk oils (Table 1) increased. These results suggest that the transfer of resveratrol from the aqueous phase into the oil phase improves the polyphenol accumulation in the protein membrane at the oil surface. Therefore, there is about 50% resveratrol at the interface of peppermint oil emulsions (Figure 5). Although the polyphenol solubility in MCT was greater than that in sunflower oil (Table 1), the oily percentages of resveratrol in MCT emulsions are similar to those in sunflower oil emulsions (Figure 6). It is suggested that the transfer of resveratrol from the aqueous phase into the MCT phase was withheld by the interfacial proteins, due to the complexation or encapsulation by proteins. When curcumin was added from the oily phase at the polyphenol concentration below its solubility in MCT, β-lactoglobulin at the oil–water interface of emulsions had a better capability of lowering the interfacial tension compared with protein alone, suggesting that the curcumin could accumulate at the protein layer at the interface [41]. Moreover, the curcumin transfer to the oil–water interface was reported to form the polyphenol–protein complex in the soybean oil emulsion stabilized by WPI [42]. Therefore, the oil–water interface provides the microenvironment for the enrichment of resveratrol by proteins (Figure 5).

## 4. Conclusions

The solubility of resveratrol was ranked in order of peppermint oil > MCT > fish oil > sunflower oil, while the polyphenol solubility in the aqueous solutions was ranked in the order of SC > hWPI > WPI. The interfacial percentage of proteins was dependent on oil type but not on the presence of resveratrol. The partition of resveratrol in O/W emulsions was affected by the solubility of resveratrol in oils and the interfacial protein layer. There is a negative correlation with the aqueous percentage of resveratrol but a positive correlation with the interfacial percentage of resveratrol with the percentage of interfacial protein and the polyphenol solubility in bulk oils. The difference in the partition of resveratrol and proteins at the oil–water interface increased as the polyphenol solubility in bulk oils increased. The interfacial protein layer was a barrier for the transfer of resveratrol from the aqueous phase into the inner oil phase in emulsions. Resveratrol solubility in the oil phase drives its transfer from the aqueous phase into the oil phase in all emulsions, except that the interfacial protein also contributes to the transfer in fish oil emulsions. The prediction of resveratrol partition in emulsions should be useful for the understanding of the loading of bioactive components in O/W emulsions.

## Figures and Tables

**Table 1:** Solubility of resveratrol in sunflower, fish and peppermint oils and medium-chain triglyceride (MCT) and dielectric constant of the oils.

Oil Type	Fish Oil	Sunflower Oil	MCT	Peppermint Oil
Solubility (mg/g)	0.25 ± 0.05 ^c^	0.10 ± 0.01 ^c^	4.20 ± 0.30 ^b^	15.85 ± 3.90 ^a^
Dielectric constant	3.34 ± 0.02 ^c^	3.27 ± 0.10 ^cd^	3.77 ± 0.00 ^b^	6.80 ± 0.05 ^a^

Different letters mean significant differences at *p* < 0.05.

**Table 2:** Multiple linear regression analysis of the percentages of resveratrol in the aqueous phase of fish oil, sunflower oil, MCT and peppermint oil emulsions.

Variable Description	R^2^	Regression Coefficient	*p*	β
Constant	0.806	93.237	0.000	
Resveratrol solubility in bulk oil		−0.003	0.000	−0.977
Resveratrol solubility in protein solution		0.012	0.634	0.050
Protein concentration in emulsion		−3.272	0.453	−0.101
Interface protein percentage		−1.110	0.000	−0.606
Constant	0.803	88.295	0.000	
Resveratrol solubility in bulk oil		−0.003	0.000	−0.944
Interface protein percentage		−1.018	0.000	−0.556

**Table 3:** Multiple linear regression analysis of the percentages of resveratrol in the oil phase of fish oil, sunflower oil, MCT and peppermint oil emulsions.

Variable Description	R^2^	Regression Coefficient	*p*	β
Constant	0.786	−30.692	0.000	
Resveratrol solubility in bulk oil		0.003	0.000	1.094
Resveratrol solubility in protein solution		−0.022	0.282	−0.120
Protein concentration in emulsion		10.514	0.004	0.429
Interface protein percentage		0.837	0.000	0.607
Constant	0.779	−30.637	0.000	
Resveratrol solubility in bulk oil		0.003	0.000	1.081
Protein concentration in emulsion		7.994	0.003	0.327
Interface protein percentage		0.808	0.000	0.586

**Table 4:** Multiple linear regression analysis of the percentages of resveratrol in the oil phase of sunflower oil, MCT and peppermint oil emulsions.

Variable Description	R^2^	Regression Coefficient	*p*	β
Constant	0.918	−6.606	0.401	
Resveratrol solubility in bulk oil		0.003	0.000	0.994
Resveratrol solubility in protein solution		−0.007	0.685	−0.035
Protein concentration in emulsion		2.220	0.522	0.077
Interface protein percentage		0.220	0.202	0.113
Constant	0.912	2.114	0.079	
Resveratrol solubility in bulk oil		0.002	0.000	0.955

**Table 5:** Multiple linear regression analysis of the percentages of resveratrol at the oil–water interface of fish oil, sunflower oil, MCT and peppermint oil emulsions.

Variable Description	R^2^	Regression Coefficient	*p*	β
Constant	0.626	37.433	0.000	
Resveratrol solubility in bulk oil		0.000	0.006	0.367
Resveratrol solubility in protein solution		0.010	0.516	0.096
Protein concentration in emulsion		−7.237	0.007	−0.527
Interface protein percentage		0.273	0.020	0.353
Constant	0.521	21.351	0.000	
Resveratrol solubility in bulk oil		0.001	0.000	0.597
Interface protein percentage		0.533	0.000	0.690

**Table 6:** Multiple linear regression analysis of the percentages of resveratrol at the oil–water interface of fish oil, MCT and peppermint oil emulsions.

Variable Description	R^2^	Regression Coefficient	*p*	β
Constant	0.792	30.870	0.000	
Resveratrol solubility in bulk oil		0.001	0.000	0.618
Resveratrol solubility in protein solution		0.002	0.902	0.016
Protein concentration in emulsion		−5.518	0.018	−0.395
Interface protein percentage		0.364	0.001	0.495
Constant	0.715	17.146	0.000	
Resveratrol solubility in bulk oil		0.001	0.000	0.839
Interface protein percentage		0.566	0.000	0.768

## Data Availability

Data are contained within the article or Supplementary Material.

## Supplementary Materials

The following supporting information can be downloaded at https://www.mdpi.com/article/10.3390/antiox12030589/s1, Figure S1: The aqueous phase and the emulsified oil droplets of O/W emulsions where the emulsified oil droplets include the interfacial protein membrane and the inner oil phase and spatial partition of resveratrol in the emulsion; Figure S2: Interfacial percentage of native WPI (A), heat-denatured WPI (B) and sodium caseinate (C) in sunflower, fish, MCT and peppermint oil emulsions without and with 130 μg/mL resveratrol at the protein content of 1%. Different letters on top of the rectangle mean significant differences at *p* < 0.05; Figure S3: (A) Interfacial protein replacement of fish oil emulsions made with native WPI, heat-denatured WPI (hWPI), sodium caseinate (SC) using 3% Tween 20 under votexing for 30 s without and with stirring for 2 h. (B) Interfacial replacement of WPI, hWPI and SC in fish oil, sunflower oil, MCT and peppermint oil emulsions under votexing for 30 s. The concentrations of proteins are 0.5% (A) and 2% (B) in emulsions. Different letters mean significant differences at *p* < 0.05; Figure S4: Percentage of free resveratrol of the aqueous phase of fish oil, sunflower oil and MCT emulsions stabilized by 2% WPI, hWPI and SC. Different letters mean significant differences at *p* < 0.05; Figure S5: Correlation between experimental and predicted resveratrol percentages in the aqueous phase (A), oil phase (B) and interface (C) of fish oil, sunflower oil, MCT and peppermint oil emulsions and in the oil phase (D) of sunflower oil, MCT and peppermint oil emulsions and the interface (E) of fish oil, MCT and peppermint oil emulsions by the regression models; Table S1: Pearson correlation coefficient (*r*) between the added concentration of proteins in emulsions and the interfacial protein percentages.
