# Supplementary material for: Effect of Oil Type on Spatial Partition of Resveratrol in the Aqueous Phase, the Protein Interface and the Oil Phase of O/W Emulsions Stabilized by Whey Protein and Caseinate
Source: Antioxidants (Basel). 2023 Feb 27;12(3):589. doi: 10.3390/antiox12030589 (PMC10045782; doi:10.3390/antiox12030589)
Supplement: Supplementary file 1 [file antioxidants-12-00589-s001.zip › antioxidants-2234411-supplementary.pdf]

Supplementary data

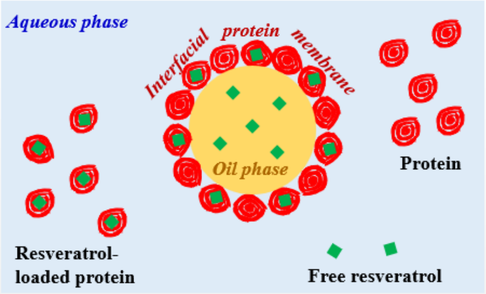

Figure S1. The aqueous phase and the emulsified oil droplets of O/W emulsions where the emulsified oil droplets include the interfacial protein membrane and the inner oil phase and spatial partition of resveratrol in the emulsion.

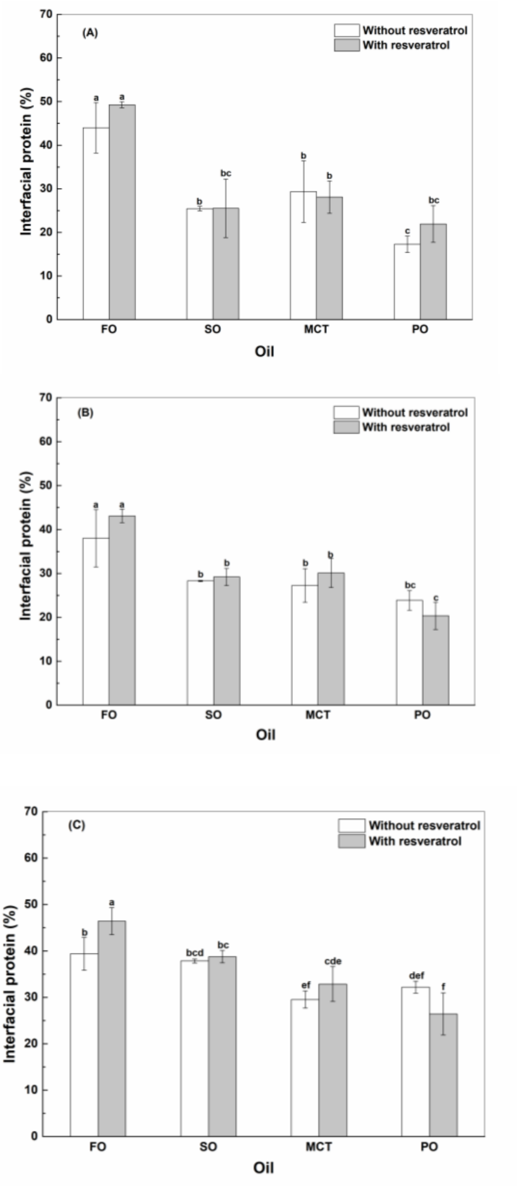

Figure S2. Interfacial percentage of native WPI (A), heat-denatured WPI (B) and sodium caseinate (C) in sunflower, fish, MCT and peppermint oil

emulsions without and with 130 g/mL resveratrol at the protein content of 1%. Different letters on top of the rectangle mean significant differences at $p < 0.05$.

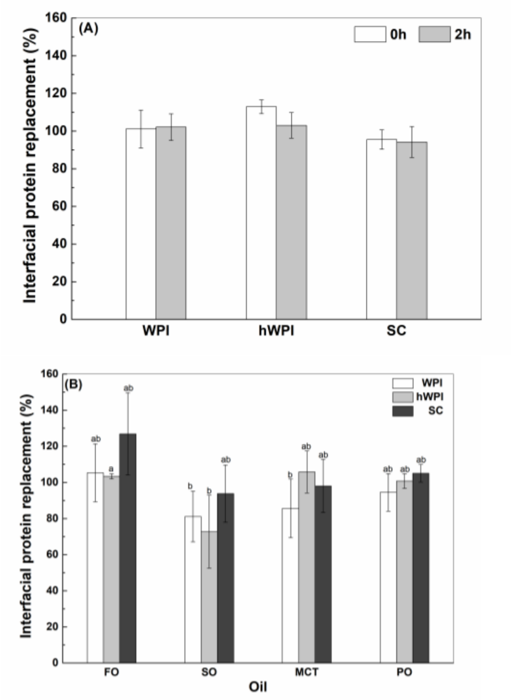

Figure S3. (A) Interfacial protein replacement of fish oil emulsions made with native WPI, heat-denatured WPI (hWPI), sodium caseinate (SC) using 3% Tween 20 under votexing for 30 s without and with stirring for 2 h. (B) Interfacial replacement of WPI, hWPI and SC in fish oil, sunflower oil, MCT and peppermint oil emulsions under votexing for 30 s. The concentrations of proteins are 0.5% (A) and 2% (B) in emulsions. Different letters mean significant differences at $p < 0.05$.

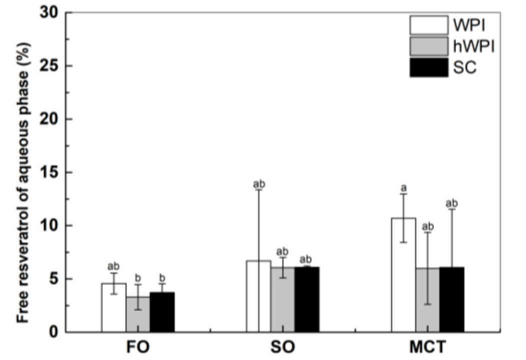

Figure S4. Percentage of free resveratrol of the aqueous phase of fish oil, sunflower oil and MCT emulsions stabilized by 2% WPI, hWPI and SC. Different letters mean significant differences at $p < 0.05$.

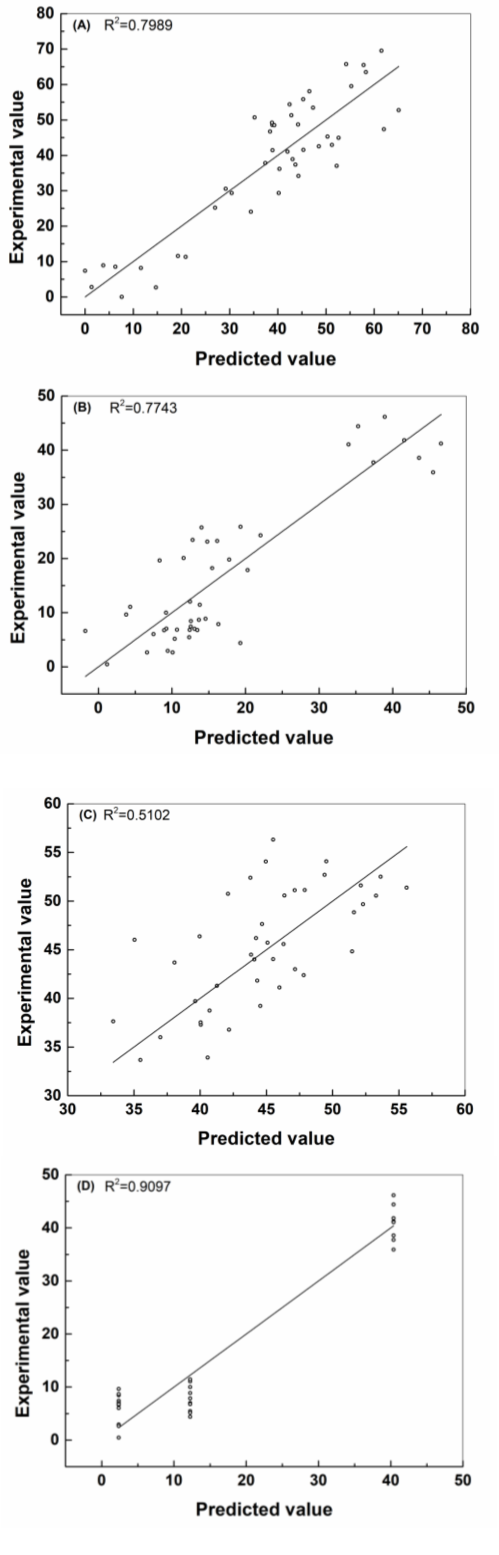
(A) R2=0.7989
(B) R2=0.7743
(C) R2=0.5102
(D) R2=0.9097
Experimental value
Predicted value

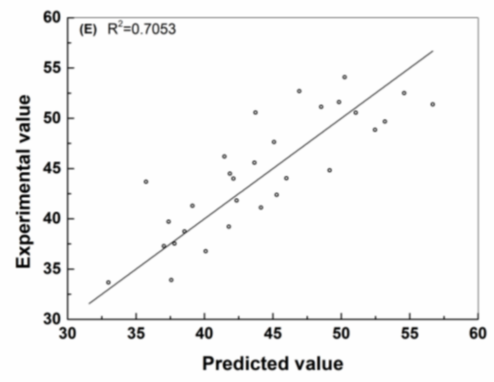

Figure S5. Correlation between experimental and predicted resveratrol percentages in the aqueous phase (A), oil phase (B) and interface (C) of fish oil, sunflower oil, MCT and peppermint oil emulsions and in the oil phase (D) of sunflower oil, MCT and peppermint oil emulsions and the interface (E) of fish oil, MCT and peppermint oil emulsions by the regression models.

Table S1. Pearson correlation coefficient (r) between the added concentration of proteins in emulsions and the interfacial protein percentages

Parameter	Oil type			
	Fish oil	Sunflower oil	MCT	Peppermint oil
r	-0.852	-0.795	-0.753	-0.869
p	0.000	0.002	0.005	0.002
